# Supplementary material for: Evaluation of the Antifungal, Antioxidant, and Anti-Diabetic Potential of the Essential Oil of Curcuma longa Leaves from the North-Western Himalayas by In Vitro and In Silico Analysis
Source: Molecules. 2022 Nov 8;27(22):7664. doi: 10.3390/molecules27227664 (PMC9695312; doi:10.3390/molecules27227664)
Supplement: Supplementary file 1 [file molecules-27-07664-s001.zip › molecules-1985903-supplementary.pdf]

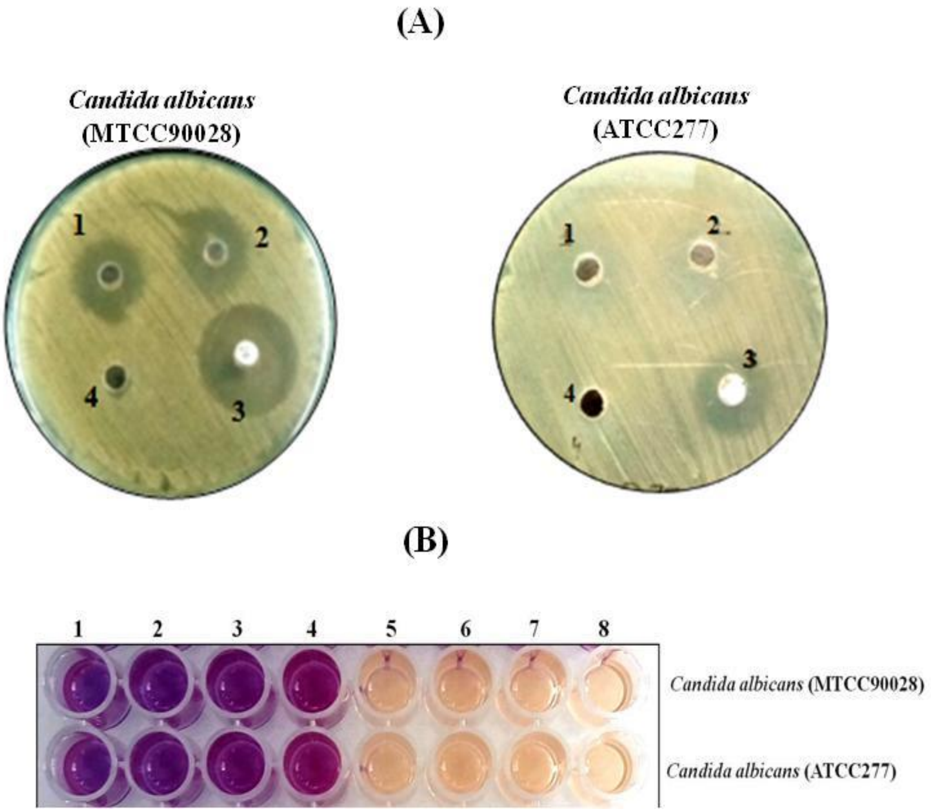

Figure. S1: Antifungal activity of CLO. A) Agar well diffusion assay. B) Broth dilution method. In agar well diffusion method, 1 represent 25 μL of CLO, 2 represent 50 μL of CLO, 3 represent positive control, fluconazole, 4 represent negative control, DMSO. In broth dilution assay, purple color of dye indicates fungal growth, while pink color showed no growth of fungal culture.

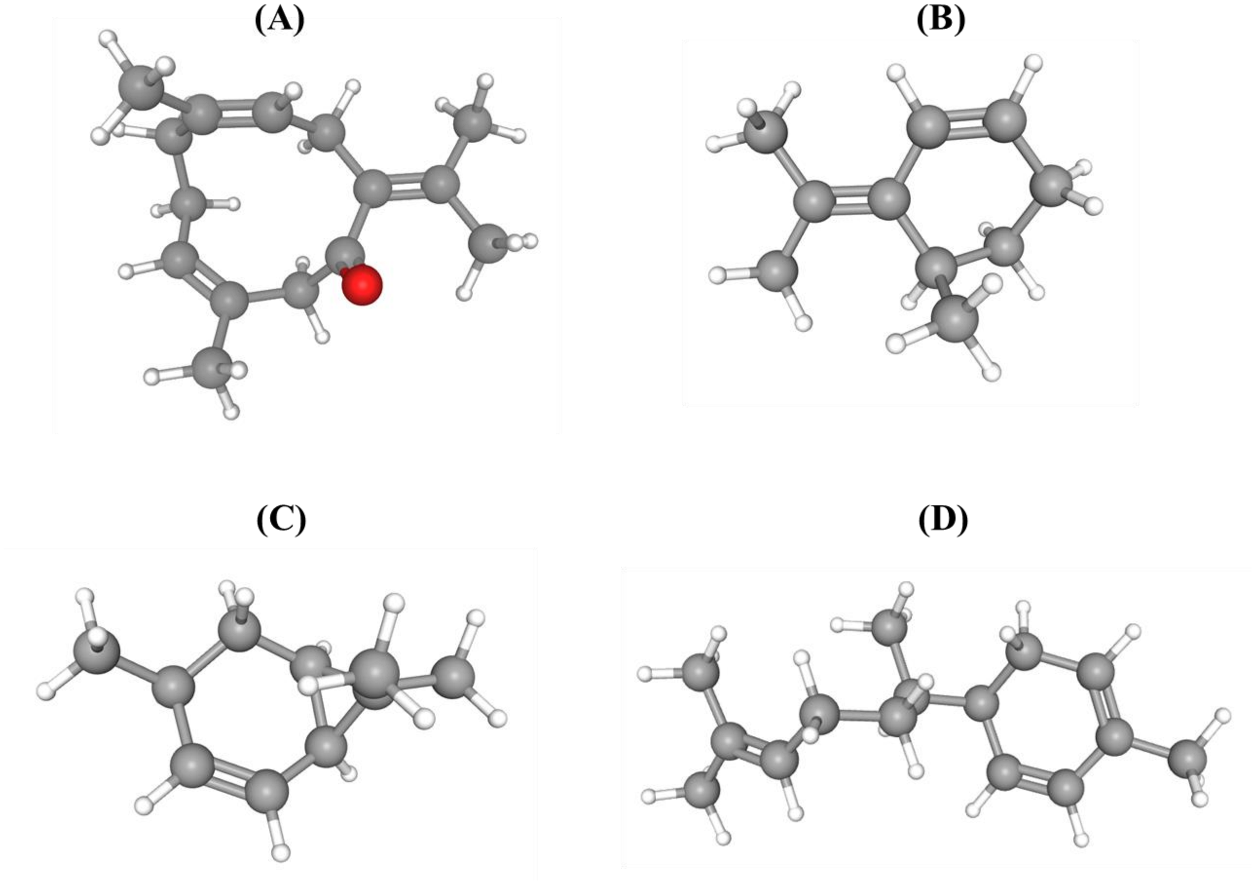

Figure S2: 3-D structure of major phytocompounds identified in GC-MS analysis of CLO selected for docking. 3,7-Cyclodecadien-1-one, 3,7-dimethyl-10-(1-methylethylidene) (A), Cyclohexene, 4-methyl-3-(1-methylethylidene) (B), (+)-4-Carene (C), 1, 3-Cyclohexadiene (D).

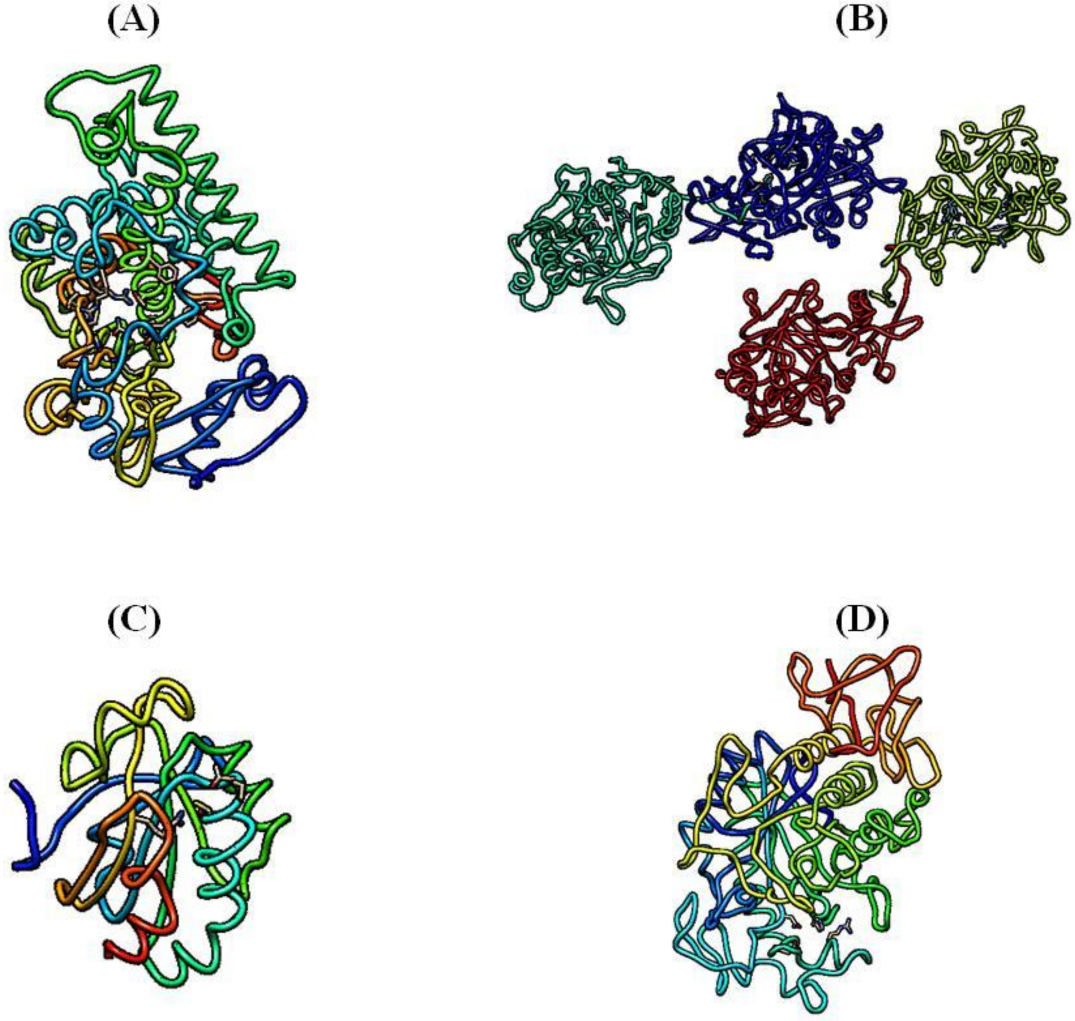

Figure S3: Target proteins selected as receptors for docking of phytocompounds of CLO. Crystal structure of cytochrome P_{450} 14 alpha–sterol demethylase cytochrome P_{450} 14 alpha–sterol demethylase (CYP51; PDB ID: 1EA1) (A), N-myristoyl transferase (NMT; PDB ID: 1IYL) of *C. albicans* (B), Human peroxiredoxin 5 (PDB ID: 1HD2) (C), and Human pancreatic alpha-amylase (PDB ID: 1HNY) (D).
